# Reducing Disparities in the Burden of Cancer: The Role of Patient Navigators

**DOI:** 10.1371/journal.pmed.0030193

**Published:** 2006-07-11

**Authors:** Tisha Fowler, Caryn Steakley, A. Roland Garcia, Jennifer Kwok, L. Michelle Bennett

## Abstract

Many racial and ethnic minority patients with cancer face barriers related to access to health care and information. Patient navigators, say the authors, could help to overcome these barriers.

Cancer incidence and death rates for all cancers combined decreased from 1991 through 2001 [ 1]. However, when these data are examined by race/ethnicity or socioeconomic status, significant differences emerge. The National Cancer Institute (NCI) defines cancer health disparities as “differences in the incidence, prevalence, mortality and burden of cancer and related adverse health conditions that exist among specific population groups in the United States.” Disparities extend beyond race/ethnicity and gender and include age, socioeconomic status, disability, geographic location, and sexual orientation [ 2].

Whereas many questions remain as to what causes cancer health disparities, the 2001 President's Cancer Panel report clearly documents that many racial and ethnic minorities as well as other underserved populations face a number of barriers related to access to health care and information [ 3]. The Institute of Medicine's *Unequal Treatment: Confronting Racial and Ethnic Disparities in Health Care* further confirmed that even with comparable insurance coverage, minorities experienced an array of additional barriers to care, such as language and cultural familiarity [ 4].

Racial and ethnic minorities are also underrepresented in clinical trials, which makes it harder to generalize trial results to minority groups. A recent *PLoS Medicine* study found that minority patients were less likely to be invited to participate in trials, and that when minority patients were informed of trials and invited to join, they were just as willing to participate as non-minority patients [ 5]. The authors of the study concluded that efforts should be made to provide better access for minority patients in clinical trials by addressing barriers to their participation (e.g., eligibility requirements, child care, and reimbursement of travel expenses). One potential strategy to directly improve access to health-care information and opportunities to participate in clinical trials among minority and other underserved populations is through the use of patient navigators. In this article, we describe one of NCI's initiatives on patient navigation and its potential role in reducing cancer health disparities.

## What is Patient Navigation?

While there is a growing interest in patient navigation, there is no consensus as to what patient navigation is or who patient navigators actually are. Because of this lack of standardization, a recent literature review recommended that a navigator be defined as “someone who helps assist patients overcome barriers to care,” instead of trying to use a more service-based definition [ 6]. Patient navigation refers to the assistance offered to underserved populations in “navigating” through the complex health-care system to overcome barriers in accessing quality care and treatment. Patient navigation is intended to help patients with cancer overcome obstacles to timely diagnosis and treatment [ 7].

Historically, the concept of patient navigation can be attributed to Harold Freeman, a distinguished cancer surgeon and former president of the American Cancer Society (1988–1989). In 1990, Freeman and colleagues implemented the nation's first patient navigator program at Harlem Hospital in New York. The purpose of the program, sponsored by the American Cancer Society, was to assist low-income and medically underserved patients in overcoming barriers to obtaining prompt diagnostic and treatment services following abnormal or suspicious cancer screening findings [ 8]. A program evaluation showed that patients who received patient navigator services had a significantly decreased time until follow-up services were received compared to those who did not. Similarly, a later study at Harlem Hospital found significant improvements in diagnosis and five-year survival rates among patients with breast cancer and attributed the improvement to patient navigation services [ 7].

## The NCI's Patient Navigator Academy

In recognition of the fact that patient navigators can play an important role in promoting access to clinical trials, the NCI's Center for Cancer Research, in collaboration with the Center to Reduce Cancer Health Disparities and the Division of Cancer Treatment and Diagnosis, sponsored the first NCI Patient Navigator Academy (PNA) in the spring of 2005 at the newly opened National Institutes of Health Clinical Research Center in Bethesda, Maryland. This three-day program provided patient navigators with an overview of cancer, its treatment and psychosocial impact on individuals, and an overview of clinical trials and resources available for access to cancer clinical trials, both at the National Institutes of Health Clinical Center and nationally.

For this first workshop, we invited existing patient navigators from the NCI-supported Cancer Disparities Research Partnership program and two local hospitals. These navigators represented patients in a wide range of racial and ethnic populations from diverse geographic locations, including Native Americans, African Americans, and Hispanic/Latino communities from both rural and urban areas, as well as distinct communities such as the Amish. Prior to the workshop, phone interviews were held with the participants to discuss the topics that would be most relevant to their needs and to learn about specific difficulties to cancer care among their patient populations. The navigators identified the following barriers and challenges: transportation, insurance coverage, patient cultural and religious beliefs, and establishing trust within the local community. This information was used to establish a framework for the workshop agenda ( Box 1) and for establishing the training objectives ( Box 2). Highlights of the workshop are detailed in Box 3.

## Lessons Learned from the First Patient Navigator Academy

Because navigators work in a variety of institutions and with distinct patient populations, providing information that is relevant to each setting is a challenge. However, developing an educational module to broadly address cross-cutting issues faced by many of the navigators (such as insurance coverage and transportation) may be an effective way to stimulate discussion among participants at future workshops. Such a module might also be the start of a process of identifying best practices for patient navigation. The module could then be tailored to meet the cultural, spiritual, and language needs of different communities.

The PNA workshop provided patient navigators from across the country an opportunity to communicate and build a network of contacts with one another. While the PNA was successful at building a foundation, there remains a need to establish a mechanism or venue for ongoing interaction and communication for patient navigators to learn from one another and share their experiences. Lastly, patient navigators stressed an interest in program sustainability. Herein remains an opportunity for educating patient navigators on potential funding mechanisms in addition to advocacy techniques.

## The Future of Patient Navigation and Recent Initiatives

In June 2005, the Patient Navigator Outreach and Chronic Disease Act of 2005 was enacted into public law in the United States [ 9]. An amendment to the Public Health Service Act, this law authorizes the Health Resources and Services Administration to administer a US$25 million demonstration grant program. This program is designed to provide patient navigator services with the goal of reducing barriers and improving health-care outcomes. Funded entities will be required to recruit, train, and employ patient navigators to provide services to health disparity populations. This act requires coordination among the Indian Health Service, the Office of Rural Health Policy, the NCI, and other needed agencies and offices in the design and evaluation of the program.

Despite the disagreement on the conceptualization of patient navigation, researching its effectiveness within the cancer care system is a focus of NCI's Patient Navigator Research Program. The program describes patient navigation as supporting and guiding the patient with cancer and/or the patient's family from the time of abnormal finding to the completion of cancer treatment [ 10]. The roles and responsibilities of patient navigators extend beyond scheduling appointments and coordinating insurance to include community education and outreach, forming partnerships, and encouraging clinical trials participation. Research focused on evaluating the effectiveness of patient navigation will provide data to determine if patient navigator programs can reduce cancer health disparities.

**Box 1. PNA Major Topic Areas**

- Cancer overview
- Clinical trials overview
- Human subjects protection
- Roles and responsibilities of the research team
- Insurance and clinical trials
- Recruitment to clinical trials
- Finding oncology clinical trials
- Health disparity patient navigation
- Ethical considerations in oncology clinical trials

**Box 2. Workshop Objectives**

- Understanding NCI's perspective on patient navigation and health disparities
- Recognizing the common classification systems for cancer
- Discussing cancer development
- Understanding cancer treatments
- Discussing the psychosocial impact of cancer
- Defining clinical trials and identifying the different types of trials
- Understanding how to access and enroll in clinical trials

**Box 3. The First NCI PNA**

During the workshop clinical staff and science professionals presented detailed lectures and encouraged interactive participation. To supplement the didactic course material, Harold Freeman addressed the group, providing a history of navigation and his vision. Navigators also toured the National Institutes of Health Clinical Center and shadowed a research nurse from the Center for Cancer Research to experience firsthand how the clinical trials process occurs within the NCI intramural program. An expert panel addressed issues of palliative care, complementary and alternative medicine, and cancer education resources, particularly as they related to underserved and minority populations. The workshop concluded with the navigators' case studies and group discussion. During this exchange navigators emphasized that being open and honest is absolutely essential to building and nurturing trust with their communities. Other elements that emerged as critical for a successful program included having strong administrative and physician support, ensuring that initial contact with the patient occurs at the earliest point possible, and empowering patients to make informed decisions by ensuring they have access to appropriate information.

## Abbreviations

NCI: National Cancer Institute
PNA: Patient Navigator Academy
